# FES null mice demonstrate a reduction in neutrophil dependent pancreatic cancer metastatic burden

**DOI:** 10.3389/fonc.2023.1096499

**Published:** 2023-03-09

**Authors:** Jan E. Strøbech, Pietro Giuriatti, Rikke Stagaard, Paulo De Sepulveda, Sebastian R. Nielsen, Janine T. Erler

**Affiliations:** ^1^ Biotech Research and Innovation Center (BRIC), University of Copenhagen (UCPH), Copenhagen, Denmark; ^2^ Cancer Research Center of Marseille (CRCM), Institut National de la Santé et de la Recherche Médicale (INSERM), Centre National de la Recherche Scientifique (CNRS), Aix-Marseille University, Institute Paoli-Calmettes, Marseilles, France

**Keywords:** PDAC – pancreatic ductal adenocarcinoma, neutrophils, FES, metastatic burden, liver metastasis, cancer

## Abstract

Patients with pancreatic ductal adenocarcinoma (PDAC) have a dismal 5-year survival rate of less than 10%, predominantly due to delayed diagnosis and a lack of effective treatment options. In the PDAC tumor microenvironment (TME), neutrophils are among the immune cell types that are most prevalent and are linked to a poor clinical prognosis. However, treatments that target tumor-associated neutrophils are limited despite recent developments in our understanding of neutrophil function in cancer. The feline sarcoma oncogene (FES) is a nonreceptor tyrosine kinase previously associated with leukemia and hematopoietic homeostasis. Here we describe a newly derived FES null mouse with no distinct phenotype and no defects in hematopoietic homeostasis including neutrophil viability. The immune cell composition and neutrophil population were analyzed with flow cytometry, colony-forming unit (CFU) assay, and a neutrophil viability assay, while the response to PDAC was examined with an *in vivo* cancer model. In an experimental metastasis model, the FES null model displayed a reduced PDAC hepatic metastatic burden and a reduction in neutrophils granulocytes. Accordingly, our results indicate FES as a potential target for PDAC TME modulation.

## Introduction

PDAC is a cancer evolving from the lining of the glandular tissue in the pancreas. It is a relatively rare cancer, but the incidence is rising mainly, but not limited to, an aging population and lifestyle diseases (1). Although PDAC only accounts for 3.2% of all cancer cases in the U.S it is still estimated to kill almost 50.000 people surpassing breast cancer in lethality despite breast cancer accounting for 5 times as many cancer cases in the US (2). Several factors give rise to the high lethality of this disease, including difficulties with early diagnosis, local invasion of the primary tumor to large vessels and the spine, and very early dissemination of cancer cells. As a result of the latter, surgical treatment is only applicable for less than 10% of the patients (3, 4). Treatment of PDAC is further obstructed by excessive extracellular matrix (ECM) deposition and an immunosuppressive TME (4, 5). Due to the above-mentioned complications, the overall 5-year survival rate is less than 10% (1).

The PDAC TME is highly complex and consists of stromal cells, including subpopulations of fibroblasts and infiltrating immune cells (5). The two immune cell types most prevalent in the TME are neutrophils and macrophages. Both cell types have been associated with a poor prognosis due to secreted cytokines, introduced chemoresistance, and other immunomodulatory capabilities (6). Neutrophils account for the majority of immune cells and are an important part of the innate immune response (7). Recently, there has been an increased focus on neutrophils and their double-edged nature in a cancerous setting (8, 9). A high neutrophil to lymphocyte ratio has long been a poor prognostic marker for patient survival and has dictated treatment regimens (10, 11). Neutrophils have been implicated to play a role in the establishment of the metastatic niche and are involved in rearranging the ECM (7, 12).

We have previously shown that treating mice with Lorlatinib reduced primary tumor growth and metastasis in pancreatic cancer through effects on neutrophils (13). Lorlatinib is a first-line drug for non-small cell lung carcinoma (13, 14). Our data suggested Lorlatinib inhibited the nonreceptor tyrosine kinase FES in neutrophils resulting in a reduced primary and metastatic tumor burden (13). FES has been reported to be involved in a plethora of physiological processes including survival, migration, cytokine release and hematopoietic cell differentiation (15, 16). Here, we investigated the role of FES in pancreatic cancer progression using a FES knockout (KO) mouse.

## Methods

### Animal studies

The Danish Inspectorate for Animal Experimentation authorized and provided oversight over the animal experiments. FES^-/-^ C57BL/6 mice were derived from Fes^tm2PAG^ 129X1/SvJ mice (17). FES^-/-^ mice back crossed on C57BL/6 (> 12 backcrosses) were maintained as double KO FES/FER mice. The FES mutation originates from PA Greer lab (18) and the FER from Pd Sepulveda lab (undescribed). FES^-/-^, FES^+/-^ and FES^+/+^ mice were then bred at the University of Copenhagen on a C57BL/6 background. Male and female FES^-/-^ and FES^+/+^ C57BL/6 mice, age 8 – 16 weeks old, were used for all experiments.

### Genotyping

Ear pieces from FES^-/-^, FES^+/-^ and FES^+/+^ mice were collected and lysed (50 mM KCL, 50 mM Tris HCl, 2,5 mM EDTA pH 8, 0,45% IGEPAL, 0,45% Tween 20, 10 mg/mL proteinase K) over-night at 55°C and 650 rpm. Feline sarcoma-related protein (FER) genotyping was done with *Taq* DNA Polymerase (Qiagen 201203) according to manufactures instructions. For FES genotyping Phire Tissue Direct PCR Master Mix (ThermoFisher Scientific, F170S) was prepared according to the manufactures instructions. Primers were ordered from TAQ Copenhagen. A list of primers and PCR programs can be found in the **Supplemental Materials**.

### Cell culture

Murine pancreatic cancer cells KPC mT4 were used, generated in the Tuveson Laboratory (Cold Springs Harbor Laboratory, NY, USA), isolated from PDAC primary tumors collected from *KrasLSL-G12D/+;Trp53LSL-R172H/+;Pdx1–Cre* mice with a C57BL/6 background (17). KPC mT4 cultures were cultured in DMEM + 10% FBS, 100 units of penicillin + 100 µg mL^-1^ streptomycin. Cells were tested negative from mycoplasma and murine pathogens by IMPACT testing (IDEXX Laboratories, USA).

### Conditioned medium collection

KPC mT4 cancer cells were grown to 70% confluency and then washed three times with PBS. Cells were incubated for 24h with serum-free medium before the medium was collected. Conditioned medium (CM) was filtered through a Minisart^®^ Syringe Filter (0.45µm) before use.

### Experimental liver metastasis model

1·10^5^ KPC mT4 cells were injected in the spleen of immunocompetent FES^-/-^ and FES^+/+^ C57BL/6 mice using a Hamilton 29-G syringe (19). 21 days post injection mice were weight, culled and livers were collected and weight immediately after.

### Immunohistochemistry

Mouse liver lobes were fixed in 10% neutral buffered formalin overnight at 4°C, before being embedded in paraffin according to standard protocol. Paraffin-embedded tissue was sliced to 5 µM tissue slides, mounted on glass, and then stained with hematoxylin and eosin. Stained tissue slides were scanned on a Hamamatsu NanoZoom slide scanner and metastatic lesions were quantified by size and number *via* NDP.view2 software. The metastatic index was calculated as the aggregate size of metastatic lesions as a percentage of the total area of the section.

### Neutrophil isolation and purification

#### Neutrophil viability

The femur and tibia from FES^-/-^ and FES^+/+^ C57BL/6 mice were flushed with MAC buffer (1x PBS, 0.5% wt:vol BSA, 2mM EDTA pH 8) to collect bone marrow (BM). Erythrocytes were lysed in the BM fraction with BD pharm Lyse (BD Biosciences, 555899), after which neutrophils were purified with the Neutrophil Isolation Kit (Miltenyi) according to the manufacturer’s instructions. Purified neutrophils were seeded in a concentration of 100.000 cells pr. well in poly-lysine coated 96 well plates. Neutrophils adhered for 1h in DMEM + 5% FBS before being supplementing with DMEM with either 10 ng/mL GM-CSF, 40 ng/mL G-CSF or KPC-CM (+5% FBS). Neutrophils were incubated for two days at 37°C before viability was measured with CellTiter-Glo (Promega) according to manufactures instructions and luminescence was measured on an Elisa plate reader. The luminescence signal was normalized to a measurement taken on day 0.

#### BM colony formation

BM was isolated, and Erythrocytes were lysed as described above. A triplicate of cells (3.000 pr. plate) was cultured in semi-solid methylcellulose at 37°C (MethoCult #3534; StemCell Technologies containing SCF, interleukin (IL)-3, and IL-6). Plates were analyzed after 3 days of incubation and photographed and colonies consisting of more than 10 cells were counted.

### Western blotting

Neutrophils were isolated as described above and lysed with M-PER™ Mammalian Extraction Buffer supplemented with Halt™ Phosphatase inhibitor Cocktail (1:100) and Halt™ Protease inhibitor Cocktail (1:100). Lysates were resolved on NuPAGE 4-12% Bis-Tris gels (ThermoFisher Scientific, #17080971) and blotting was done with nitrocellulose membranes (Thermo Scientific, #88018). 5% skimmed milk powder in T-BST was used as blocking and for antibody incubation. Primary antibody incubation was done overnight at 4°C and secondary antibody incubation was 1 hour at RT. Membranes were stripped using Restore™ PLUS Western Blot Stripping Buffer (ThermoFisher Scientific, #46430). Digital images of blots were prepared with ImageQuantTM LAS 400 instrument and images were analyzed with ImageJ. A list of antibodies can be found in **Supplemental Materials**.

### Flow cytometry

To prepare single cell suspensions from murine BM, the hind legs were flushed, and erythrocytes were lysed as described above. The suspension was filtered through a 40 µm cell strainer (Falcon Cell Strainer 40 µm, #352340) and blocked with FC-block (BD Pharmingen, clone 2.4G2) for 10 minutes on ice before staining with Sytox viability marker (Life Technologies) and fluorochrome-conjugated antibodies. Single-cell suspensions from metastatic murine livers were mechanically disrupted before additional mechanical and enzymatic disruption in Hanks balanced salt solution (HBSS) supplemented with 1 mg mL-1 collagenase P (Roche) on a GentleMACS Dissociator^™^ using the mTDK1 program. After dissociation cells were washed in HBSS and resuspended in 1 mL 0.05% trypsin and incubated for 5 min at 37°C before the suspension was filtered through a 70 µm cell strainer and resuspended in MAC buffer. Hepatic samples were stained with a similar regime as BM samples. A list of fluorochrome-conjugated antibodies can be found in **Supplemental Materials**.

### Statistical analysis

Student’s *t* test was used for comparing differences between two groups. For BM colony formation assay we used nested *t* test. A *P* value below 0.05 was considered significant.

## Results

### Deriving a FES null mouse with C57BL/6 background

**Figure 1:**
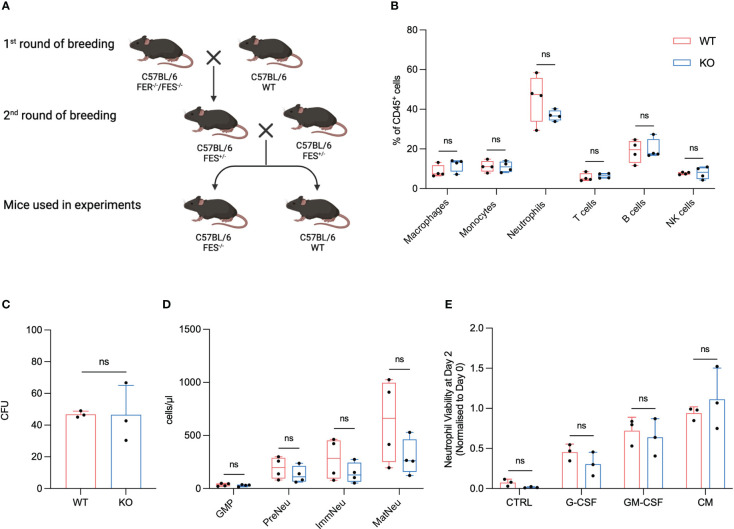
FES null mice show no alterations in immune cell composition, granulopoiesis and neutrophil viability compared to wildtype mice. **(A)**, FES/FER null mice (C57BL/6) was bred with WT C57BL/6, and the offspring subsequently backcrossed until FES^+/-^ mice were obtained. The FES^+/-^ offspring was then used for the 2^nd^ round of breeding. Only WT and FES^-/-^ offspring from 2^nd^ round of breeding was use for experiments. **(B)**, Flow cytometry analysis of immune cells from BM from healthy WT and FES^-/-^ mice (WT mice n=4, FES^-/-^ mice n=4). **(C)**, Colony-forming unit (CFU) assay of BM cells from healthy WT and FES^-/-^ mice. Colonies with >20 cells were quantified after 3 days (n=3 independent experiments). **(D)**, Flow cytometry analysis of GMPs, pre-neutrophils (PreNeu), immature (ImmNeu) and mature (MatNeu) neutrophils from BM from healthy WT and FES^-/-^ mice (WT mice n=4, FES^-/-^ mice n=4). **(E)**, quantification of neutrophil viability assay. Neutrophils were isolated from BM from either WT or FES^-/-^ mice and stimulated over two days with KPC-CM, 20 ng/mL GM-CSF or 20 ng/mL G-CSF (n = 3 independent experiments). Bar graphs represent mean and standard deviation; hypothesis testing was performed using unpaired two-sided Student’s t test. ns, Non significant

To further explore the FES kinase and its role in metastatic pancreatic cancer, we used a FES KO mouse model (18). We derived the FES single KO by crossing the double KO with wild-type (WT) C57BL/6 mice and backcrossing the offspring (**Figure 1A**). Breeding was confirmed *via* PCR (data not shown) and FES KO validated by western blotting (**Figures S1A, B**).

To our knowledge a characterization of a FES KO with C57BL/6 background have not been described. FES homozygote KO were bred with heterozygotes FES^+/-^ pairs. Offspring of the heterozygote breeding pairs kept the mendelian ration and no stunting or abnormalities was observed in the litters.

To investigate if any alterations in the immune cell fractions could be observed between the FES KO and the WT littermates, we isolated BM from healthy WT and KO mice and analyzed samples using flow cytometry. There were no significant differences observed in numbers of F4/80^+^ macrophages, Ly6C^+^ monocytes, Ly6G^+^ neutrophils, CD3^+^ T cells, B220^+^ B cells or NK1.1^+^ NK cells between WT and FES KO littermates (**Figure 1B**).

Our previous results suggest that granulopoiesis is hampered in healthy mice when they are treated with Lorlatinib (13). To examine if a similar discrepancy could be observed between FES null mice and WT, we isolated BM from healthy KO and WT mice to assess its ability to establish colonies in semisolid methylcellulose medium with recombinant cytokine supplement (IL-3, IL-6 and SCF). Freshly isolated BM cells were incubated in methylcellulose over a three-day period before counting of the subsequent colonies. We observed no significant changes between WT and KO mice regarding number of colonies suggesting that FES is not directly involved in granulopoiesis in our model and that Lorlatinib could potentially have some off-target effects (**Figure 1C**). To further explore this finding, we isolated BM from WT and FES KO mice and analyzed neutrophil subpopulations using flow cytometry. Consistent with the granulopoiesis assay, we did not observe any significant changes between WT and FES KO mice with regards to CD16/32^hi^ granulocyte-monocyte progenitor cells (GMPs), cKit^+^ pre-neutrophils, Ly6G^+^/CXCR2^-^ immature neutrophils and Ly6G^+^/CXCR2^+^ mature neutrophils underlining that the FES kinase is not involved in neutrophil development in steady state conditions (**Figure 1D**).

We have previously showed that FES is primarily expressed in neutrophils (13), thus we isolated neutrophils from the BM of healthy KO and WT mice to assess the viability of neutrophils over a three-day period. According to previous studies, neutrophil viability rapidly decreases in standard cell culture medium and is undetectable by day 2 (19). However, when neutrophils were grown in DMEM and supplemented with either KPC-CM, GM-CSF, or G-CSF, we were able to restore neutrophil viability. Importantly, no difference in neutrophil viability was observed between WT and FES KO cells (**Figure 1E**).

Taken together, these results show that mutation of the *fes* gene in mice with C57BL/6 background does not affect the viability of the offspring and makes FES KO mice indistinguishable from WT littermates with regards to immune cell composition, neutrophil development and neutrophil viability.

### FES KO mice show reduced metastatic burden

As mentioned earlier, the vast majority of PDAC patients either have unresectable tumors or metastatic disease, with the liver as the primary target for cancer cells to migrate to, lowering the survival rate substantially compared to patients with resectable tumors (20, 21).

**Figure 2:**
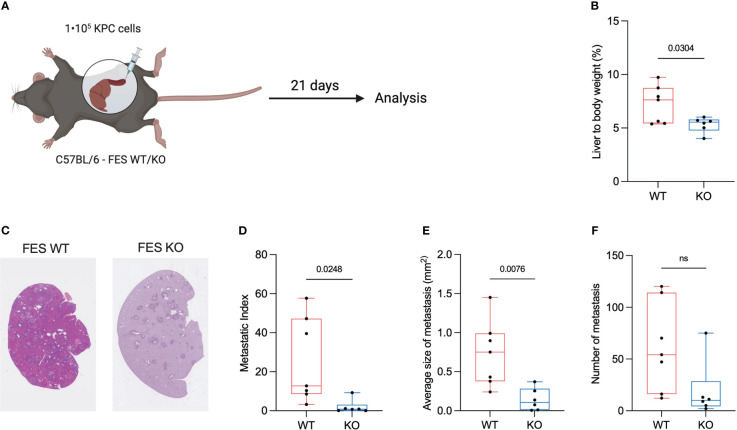
FES null mice attenuate progression of hepatic PDAC metastasis. **(A)**, Schematic showing intrasplenic implantation of KPC mT4 cancer cells. **(B)**, Liver weight (%) of total body weight WT and FES null mice with metastatic disease. (WT mice n=7, FES null mice n=6). **(C)**, Representative hematoxylin & eosin stainings from FES WT and FES KO mice **(D)**, Metastatic index calculated as a percentage of metastatic lesions from hematoxylin and eosin stained liver section in WT and FES null mice. (WT mice n=7, FES null mice n=6). **(E)**, Quantification of average size of metastatic lesions from hematoxylin and eosin stainings (WT mice n=7, FES null mice n=6). **(F)**, Number of metastatic lesions per liver after intrasplenic injection (WT mice n=7, FES null mice n=6). Data in c – f are from two independent experiments. Bar graphs represent mean and standard deviation; hypothesis testing was performed using unpaired two-sided Student’s t test. ns, Non significant

To determine the FES kinase’s involvement in liver metastasis formation, we implanted KPC pancreatic cancer cells in healthy FES null mice and their WT littermates. KPC cells were injected in the distal part of the spleen where they migrate through the portal vein and establish metastases in the liver (**Figure 2A**). 21 days post injection the mice were euthanized and analyzed.

A feature of PDAC tumors is excessive ECM deposition, which is thought to reduce the effectiveness of treatment by limiting drug delivery (22, 23). Excessive desmoplasia could result in an increase in the liver to bodyweight ratio and we hypothesize that an increase in liver to bodyweight ratio is connected to an increased ECM deposition. To assess if either WT or KO mice had an increased liver to body ratio, livers were weight immediately after euthanization. We found that the ratio was significantly reduced in FES KO mice compared to WT (**Figure 2B**).

To assess metastasis formation liver sections were H&E stained (**Figure 2C**) and metastatic index as well as the size of the metastatic lesions were quantified. Both the average size of the metastatic lesions and the metastatic index were significantly reduced in the FES KO mice versus WT, while the number of metastases was not (**Figures 2D–F**).

These results suggests that the FES kinase is involved in the metastatic cascade and the metastatic load is reduced in its absence.

### Reduced metastatic burden is associated with a reduction in neutrophil response

PDAC tumors are distinguished by a TME with excessive ECM deposition and immune cell infiltration that is thought to promote tumor growth and hinder treatment (24). We have previously shown that inhibiting the FES kinase results in a significant reduction in neutrophils at both the primary tumor and the metastatic tumor sites (13). Thus, we analyzed livers of WT and FES KO mice with metastatic disease using flow cytometry to detect any changes in the immune cell composition.

**Figure 3:**
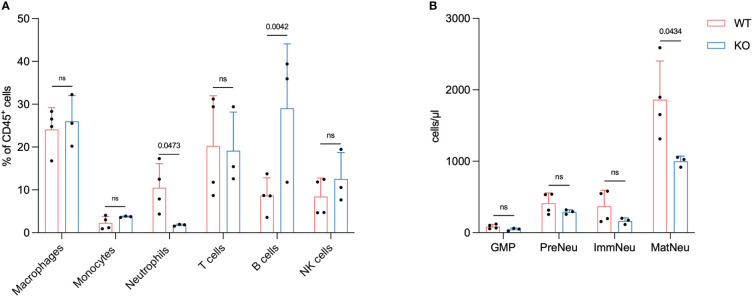
FES null mice have a reduced number of neutrophils in metastatic liver and bone marrow. Flow cytometry analysis 21 days post intrasplenic injection from mice with hepatic PDAC metastases **(A)**, Macrophages, monocytes, neutrophils, t cells, B cells and NK cells (sample from metastatic livers) (WT mice n=4, FES null mice n=3). **(B)**, GMPs, pre-neutrophils (PreNeu), immature (ImmNeu) and mature neutrophils (MatNeu) (sample from BM). Bar graphs represent mean and standard deviation; hypothesis testing was performed using unpaired two-sided Student’s t test.

After harvesting of the metastatic livers, a single cell suspension of a section of each liver was obtained and stained with appropriate antibodies. Ly6G^+^ neutrophils and B220^+^ B cells showed significant changes between the WT and the KO group. In contrast, there were no significant differences observed in F4/80^+^ macrophages, Ly6C^+^ monocytes, CD3^+^ T cells or NK1.1^+^ NK cells between WT and FES KO littermates (**Figure 3A**).

These findings align with our previous results and hypothesis that a reduction in neutrophils in metastatic liver disease reduces the metastatic progression. Previously we have shown that Lorlatinib affects neutrophil development in the BM of tumor-bearing mice (13). To test this, we isolated BM from mice with metastatic disease and analyzed the neutrophil subpopulations using flow cytometry. CD16/32^hi^ GMPs, cKit^+^ pre-neutrophils and Ly6G^+^/CXCR2^-^ immature neutrophils were not altered between WT and KO groups (**Figure 3B**). The Ly6G^+^/CXCR2^+^ mature neutrophils on the other hand showed a significant decrease in numbers, which implies that the change in neutrophils observed in the metastatic liver could be due to a reduced number of neutrophils in the BM (**Figure 3B**).

The analysis of the immune cell composition in the metastatic liver and BM of tumor bearing mice revealed that the number of mature neutrophils is reduced in KO vs. WT.

## Discussion

It is evident that patients with PDAC urgently require advancements in treatment to improve their dismal prognosis. PDAC tumors are notoriously difficult to treat and patients not eligible for surgery have few options other than palliative chemotherapy. Furthermore, the current chemotherapy regimens are decades old, and the adverse effects are legion. PDAC tumors are distinguished by a TME with excessive ECM deposition and immune cell infiltration that is thought to promote tumor growth. Consequently, the ability to alter the behavior of cells in the TME may enhance the therapeutic response in PDAC patients. Kinase inhibitors have the potential to directly target specific TME cells therapeutical and push the TME towards a more cancer-restrictive phenotype. We have previously shown by modulating neutrophils with an FES inhibitor, we affected the tumor progression and the overall survival in preclinical models.

Here we describe a KO mouse with no observable phenotype and no aberrant hematopoietic homeostasis. Specific results, previously obtained by chemically inhibiting FES in a tumor-bearing model, are reproducible in the FES null mouse confirming FES as a potential target for PDAC TME modulation.

Hackenmiller et al. characterized a FES KO mouse in a 129X1/SvJ background with notable low-penetrance defects such as embryonic lethality, cardiovascular defects, runting, skin lesions and conjunctivitis (25). Furthermore, defects in the hematopoietic system, including decreased B cell and increase in monocytes and neutrophils was also reported (25). The KO from Hackenmiller et al. was generated by substituting the sequences in the *fes* promoter region to exon 3 with a PGKneo selection cassette (25). More recently Zirngibl et al. derived a FES null mouse with a 129SvJ background by deleting the kinase domain in the *fes* gene and thus avoiding alterations on the expression of the *furin* gene located upstream of the *fes* gene (18). The *furin* product, the FUR protease, is located in the Golgi apparatus where it participates in the proteolytic maturation of a variety of other proteins (26). Alterations of FUR expression led to embryonic lethality and severe growth defects (26). These mice did not display any of the detrimental phenotypical changes seen in the previous approach, although a small reduction in circulating mature myeloid cells was observed (18). The FES/FER null mouse made available for us by Paulo De Sepulveda have a C57BL/6 background and is undescribed as far as we know. In accordance with observations by Zirngibl et al., the offspring of the newly derived FES KO mice with a C57BL/6 background were viable and showed no detrimental phenotypes. Our investigation of hematopoietic homeostasis revealed no alterations between healthy KO and WT littermates. We previously observed that granulopoiesis was altered with Lorlatinib treatment, however, as we did not observe this in the FES KO mice, it hints toward a potential effect of Lorlatinib through other targets (13). The viability of the neutrophils was unaffected by the absence of FES corresponding well with previous observations (13). Previously we have shown that treating mice with Lorlatinib during a tumor insult results in a suppression of tumor induced granulopoiesis and subsequent systemic reduction of neutrophils (13). Repeating the above mentioned experiment in our FES null mice we saw a similar reduction in neutrophils in the metastatic site and a reduction in mature neutrophils in the BM. We observed no significant decrease in immature neutrophils or GMPs in the FES null mouse. These results align with the *in vitro* granulopoiesis assay, which implies that Lorlatinib could have effects on other targets, and thus FES is not directly involved in neutrophil granulopoiesis in steady-state or under duress in our KO model.

It is important to note that inhibition and KO of a protein do not necessarily result in an identical phenotype. ATP competitor kinase inhibitors are notoriously promiscuous due to the high level of conservation in the ATP binding pocket (27). Furthermore, Lorlatinib was originally developed for ALK and ROS1 inhibition and indeed IC_50_ measurements show a hundredfold lower value for these two kinases compared to FES inhibition. However, several other kinases are also inhibited with IC_50_ values below 60 nM (14, 28). This supports the hypothesis that the inability of the KO model to mimic all the results of FES inhibition obtained with Lorlatinib, could be caused by the off-target effects of Lorlatinib.

Notably, several kinases involved in neutrophil function are reported to be inhibited by Lorlatinib. Two kinases, FAK and PTK2, that are involved in the neutrophil migration cascade were both shown to be inhibited by Lorlatinib (14, 29, 30). This could explain why the previously obtained migration results could not be reproduced in the FES KO model (data not shown). Furthermore, FES shares a distinct subgroup of protein tyrosine kinases with FER based on their common structural composition (16) and FER is also inhibited by Lorlatinib and show lower IC_50_ values than FES (14). Both FES and FER have been reported to play redundant roles despite the difference in expression patterns (31). This link between FES and FER may also be a factor in explaining our results, especially regarding granulopoiesis. Senis et al. reported that double KO (FER^-/-^/FES^-/-^) with 129X1/SvJ background showed reduced fertility and more interestingly deregulation of hematopoiesis that was not observed in the single KO models indicating a functional redundancy between FES and FER (31). This redundancy could explain the disparity in granulopoiesis between Lorlatinib inhibition of FES versus the FES null since FER, with an IC_50_ value at 3.3 nM compared to FES IC_50_ value of 6.0 nM also will be inhibited by Lorlatinib leading to a de facto double knockout (14).

In conclusion, we demonstrate that FES null mice are viable and do not exhibit a distinct phenotype with regard to immune cell fractions. Furthermore, we show that FES null mice experience a reduction in liver metastatic burden in our experimental metastasis model. This reduction in metastatic burden is accompanied by a reduction in neutrophils both at the metastatic site and in the bone marrow suggesting that FES plays a role in neutrophil development during emergency granulopoiesis. Our findings therefore confirm a role for FES in PDAC progression, and support FES targeting of neutrophils in PDAC patients through Lorlatinib treatment or development of more specific FES inhibitors.

## Data availability statement

The original contributions presented in the study are included in the article/**Supplemental Material**. Further inquiries can be directed to the corresponding authors.

## Ethics statement

The animal study was reviewed and approved by The Danish Inspectorate for Animal Experimentation.

## Author contributions

JS and SN conceived the project, JS planned the research, performed or participated in all experiments, and wrote the paper. PG performed BM colony formation, neutrophil survival and mouse genotyping. PG and RS assisted with *in vivo* experiments. PS provided FES/FER null mice and information regarding mouse genotyping. JE conceived and supervised the project and wrote the paper. All authors contributed to the article and approved the submitted version.
