# Supplementary material for: FES null mice demonstrate a reduction in neutrophil dependent pancreatic cancer metastatic burden
Source: Front Oncol. 2023 Mar 9;13:1096499. doi: 10.3389/fonc.2023.1096499 (PMC10034081; doi:10.3389/fonc.2023.1096499)
Supplement: Supplementary file 1 [file DataSheet_1.docx]

**Supplemental materials**

**Genotyping:**

3 Primers for genotyping FER:

Intron8F: GACAGTGTCTTCATTGGGTTGGCT

Intron8R: AGCTCCAGCCATGTCTTTCCCAT

GT_F: GGTCTGACGCTCAGTGGAACG

4 Primers to genotype FES:

WT1: CCAACATTGTGCGTCTCATC

WT2: CAAGTATTCCATGCAGCAG

KO1: CCGCTTCCTCGTGCTTTACGG

KO2: TAACCCACCATGTGTAGACAGG

| **PCR program** | **Temp.** | **Time** | **Cycles** |
| --- | --- | --- | --- |
| *Initial denaturation* | 98 °C | 5 min | X1 |
| *Denaturation* | 98 °C | 5 sec |  |
| *Annealing* | 56 °C | 5 sec | X35 |
| *Extension* | 72 °C | 20 sec |  |
| *Final* | 72°C | 1 min | X1 |

**Antibodies for flow cytometry:**

| **Name** | **Host** | **Clone** | **Company** | **Cat no.** | **Dilution** |
| --- | --- | --- | --- | --- | --- |
| PE/Cy7 anti-mouse CD45 | Rat | 30-F11 | Biolegend | 103114 | 1:100 |
| FITC anti-mouse/human CD11b | Rat | M1/70 | Biolegend | 101206 | 1:100 |
| PE anti-mouse F4/80 | Rat | BM8 | Biolegend | 123110 | 1:100 |
| PerCP anti-mouse Ly-6C | Rat | HK1.4 | Biolegend | 128028 | 1:100 |
| APC anti-mouse Ly6G | Rat | 1A8 | Biolegend | 127614 | 1:100 |
| PE anti-mouse CD3e | ArmenianHamster | 145-2C11 | Biolegend | 100308 | 1:100 |
| PE/Cy7 anti-mouse CD182 (CXCR2) | Rat | SA044G4 | Biolegend | 149316 | 1:100 |
| PE/Cy7 anti-mouse CD16/32 | Rat | S17011E | Biolegend | 156609 | 1:100 |
| APC anti-mouse/human CD45R/B220 | Rat | RA3-6B2 | Biolegend | 103212 | 1:100 |
| PerCP anti-mouse Nk-1.1 | Mouse | PK136 | Biolegend | 108726 | 1:100 |
| Brilliant Violet 421™anti mouse/human B220 | Rat | RA3-6B2 | Biolegend | 103239 | 1:100 |
| Brilliant Violet 421™ anti-mouse NK-1.1 | Rat | PK136 | Biolegend | 108731 | 1:100 |
| Brilliant Violet 421™ anti-mouse CD90.2 (Thy-1.2) | Rat | 53-2.1 | Biolegend | 140327 | 1:100 |
| Brilliant Violet 421™ anti-mouse I- A/I-E | Rat | M5/114.15.2 | Biolegend | 107631 | 1:100 |
| Brilliant Violet 421™ anti-mouse F4/80 | Rat | BM8 | Biolegend | 123131 | 1:100 |
| PE anti-mouse CD117 (c-Kit) | Rat | 2B8 | Biolegend | 105808 | 1:100 |
| PerCP/Cyanine5.5 anti-mouse CD115 (CSF-1R) | Rat | AFS98 | Biolegend | 135526 | 1:100 |
| PerCP-Cy™5.5 Rat Anti-Mouse Siglec-F | Rat | E50-2440 | BD Pharmingen | 565526 | 1:100 |
| APC/Cyanine7 anti-mouse Ly- 6G/Ly-6C (Gr-1) | Rat | RB6-8C5 | Biolegend | 108424 | 1:100 |
| Alexa Flour 647 Rat anti-mouse CD34 | Rat | RAM34 | BD Pharmingen | 560233 | 1:100 |

**Antibodies for western blot:**

| **Name** | **Host** | **Clone** | **Company** | **Cat. No.** | **Dilution** |
| --- | --- | --- | --- | --- | --- |
| Vinculin | Goat | hVIN-1 | Sigma | V9131 | 1:2000 |
| FES | Rabbit | D5B4Y | Cell Signaling | D5B4Y | 1:1000 |
| Anti-rabbit HRP | Goat | - | Dako | G-21234 | 1:5000 |
| Anti-mouse HRP | Rabbit | - | Dako | P0260 | 1:5000 |


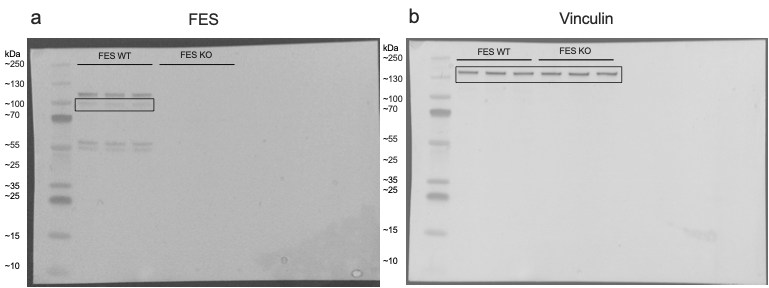


**Supplemental figure 1. Western blots from isolated neutrophil lysates. a & b.** Membrane first blotted for FES then stripped and blotted for vinculin. Each lane is one mouse. Squares indicate bands of interest.
